# The need for evidence-based strategies and tools for onchocerciasis elimination in Africa

**DOI:** 10.1186/s40249-019-0574-0

**Published:** 2019-07-15

**Authors:** Yankum Dadzie, Uche V. Amazigo, Boakye A. Boatin, Azodoga Sékétéli

**Affiliations:** 1 P. O. Box OS-1905, Accra, Ghana; 2 P. O. Box 3397, Main Post Office, Okpara Avenue, Enugu, Nigeria; 3 P.O Box CT 1380, Accra, Ghana; 4 Lomé 01, BP 3841 Togo

**Keywords:** Onchocerciasis, Elimination, Vector control, Ivermectin, Conceptual framework

## Abstract

In a recent article we discussed the feasibility of onchocerciasis elimination in Africa by 2025. We expressed concern that elimination may be impeded by failure to build on the lessons learned in the African onchocerciasis control programmes and the introduction of strategies and tools from the Americas. Richards et al. and Cupp et al. wrote to refute our concern and described recent achievements with stopping treatment in some areas.

In this response, we discuss their arguments which did not convince us. We point out several scientific flaws in the American conceptual framework of elimination which has led to longer periods of treatment than necessary, and in the use of an arbitrary threshold for stopping treatment. We show that recent achievements fall significantly short of what would be needed to achieve onchocerciasis elimination by 2025.

We conclude our response by advocating for a more objective and inclusive debate on strategies and tools for onchocerciasis elimination.

**Electronic supplementary material:**

The online version of this article (10.1186/s40249-019-0574-0) contains supplementary material, which is available to authorized users.

## Multilingual abstracts

Please see Additional file 1 for translations of the abstract into the five official working languages of the United Nations.

## Background

Richards et al and Cupp et al wrote to refute our concerns that failure to build on the lessons learned from the African onchocerciasis control programmes and the introduction of inappropriate strategies and tools from the Americas may impede progress towards achieving onchocerciasis elimination in Africa by 2025 [1, 2]. To support their argument Richards et al give an overview of the programmatic model of the Onchocerciasis Elimination Program for the Americas (OEPA) and describe several onchocerciasis projects in Africa where ivermectin treatment has recently been stopped. Cupp et al in addition to similar assertions go further to claim that OEPA’s treatment and evaluation methods are the most efficient for elimination of onchocerciasis. We are not convinced by their arguments. We would like to discuss the arguments they raise and explain the reasons for our disagreement.

## Main text

### Scientific basis and conceptual framework of elimination

Richards et al. give a lengthy description of what they call the OEPA programmatic model. They, however, do not address our critique of the scientific rationale of the OEPA conceptual framework which, in our view, is incorrect. Cupp et al. would seem to miss the point we are making. As elaborated in our article [3], two conceptual issues are the basis of onchocerciasis elimination with mass drug administration (MDA) with ivermectin:

1. Required duration of treatment to achieve elimination

The OEPA model assumes that a fixed period of 12 to 14 years of MDA with ivermectin is required to interrupt transmission and that MDA can be stopped thereafter. In this respect the OEPA framework is conceptually similar to the Onchocerciasis Control Programme in West Africa (OCP) framework for vector control that was developed and operationally used at scale by OCP in the 1980s. However, this vector control model is not valid for onchocerciasis elimination with ivermectin MDA because of the differences in the effects of vector control and ivermectin intervention on transmission and on adult onchocercal worms. Cupp et al. refer to the studies in the Americas that demonstrated partial macrofilaricidal effect of repeated ivermectin treatment on adult onchocercal worms, but this effect is not reflected in the OEPA framework. Model predictions that do take such effect into account, as well as analysis of large-scale empirical data from Africa show that the required duration of ivermectin MDA may vary from eight years of annual treatment for areas with the lowest precontrol endemicity levels to 20 years or more for areas with the highest levels of endemicity. This variation makes it important to evaluate in each project the decline in infection levels during the intervention period to ensure timely decisions as to when to stop treatment. This critical issue of impact evaluation is being overlooked since the closure of the African Programme for Onchocerciasis Control (APOC) in 2015, maybe because the imposed antibody tests cannot measure declines in active infection levels. As a result, many areas may be treated for much too long.

Annual treatment has been the principal treatment strategy for onchocerciasis control in Africa and it has also already achieved interruption of transmission and elimination of infection in several foci. However, the success of annual treatment does not exclude the use of other intervention strategies for specific purposes. The total duration of treatment could be shortened by increasing the frequency of treatment per year. Models predict that changing from annual to twice per year treatment, as recommended by Cupp et al.*,* would reduce the remaining number of years of treatment by about one third. However, this would come with an additional cost due to some 30% increase in the number of treatment rounds that would still be required [4]. The authors of that study concluded that twice yearly treatment may only be worth the effort in situations where annual treatment is expected to take a long time to achieve elimination because of unfavourable transmission conditions or because treatment started only recently. Furthermore, it is still uncertain if increasing ivermectin treatment frequency per year alone can eliminate onchocerciasis in the most intense transmission zones where additional interventions may be required. Hence there is a need to remain open-minded and evidence-based when deciding on the appropriate treatment strategy in different settings. A comprehensive review of possible interventions is provided in the report of a World Health Organization (WHO) meeting on alternative treatment strategies for onchocerciasis elimination [5].

2. Criteria for elimination and stopping treatment

As we describe in our article, OCP and APOC have eliminated onchocerciasis as a public health problem from nearly all endemic areas in Africa. APOC subsequently developed provisional criteria for stopping treatment based on model predictions and extensive empirical evidence from the OCP and APOC, and these criteria have been tested at scale in onchocerciasis foci in Mali and Senegal [6, 7]. Several CDTi projects (but no countries as yet, contrary to what Cupp et al. claim) have already been shown to meet these epidemiological criteria for elimination. The OEPA model uses the same entomological criteria as APOC but as epidemiological stopping criterion they use a prevalence of OV16 antibodies of < 0.1% in children below the age of ten years. For our reservations about the use of this serological test in terms of test characteristics (inability to detect active infection, low risk age group, sampling issues) we refer to our article [3] and other publications on the application of this serological test [8, 9]. Here we want to elaborate further on the scientific basis and selection of the 0.1% prevalence threshold.

Richards et al. state that “it was WHO Geneva, and not OEPA, which is responsible for the challenging < 0.1% threshold in children”, referring to the 2001 certification guidelines meeting report [10]. However, Cupp et al. state that the baseline document for that meeting was written by OEPA Programme Coordinating Committee members E. Cupp, R. Collins and F.Richards. Comparison of the baseline and final certification guidelines document shows that the sections relating to the 0.1% threshold, as well as most of the certification guidelines, were taken from the OEPA baseline document [11]. The baseline document does not give any scientific rationale for the 0.1% threshold but just states that it was chosen “somewhat arbitrarily”. We are concerned that since 2001 there has been no effort to put a scientific basis to this threshold which remains arbitrary to date.

The 2001 document was to serve as guidelines for national certification of elimination of human onchocerciasis (starting three to five years after cessation of treatment at the earliest). It was not, as suggested by Cupp et al.*,* a WHO endorsed global strategy for elimination. In fact it is stated upfront that “the epidemiological characteristics in Africa imply that the elaborated framework might not be technically and operationally feasible in most endemic areas of the African continent” [10]. Notwithstanding, much of the 2001 document was transported to the 2016 guidelines [12] without review and without endorsement by the Guidelines Development Committee. Although the process for developing the 2016 Guidelines followed the new international standards, as stipulated in the second edition of the WHO handbook for guideline development published in 2014, the outcome of the process was determined by the fact that the composition of the committee had been skewed in favour of OEPA. This fact thus determined the outcome of the voting on recommendations, particularly on the diagnostic tool Ov16, for which there was no consensus in the committee.

In the formation of the committee it was apparently not considered that the African Programme had in the meantime demonstrated the feasibility of elimination in Africa, has had its objective modified to address elimination and, as instructed by its board, had already started an extensive process to generate the evidence base for when and where ivermectin treatment could be safely stopped [13].

### Achievements to date and the role of the OEPA model and tools

Richard et al. claim great success with 3.8 million treatments halted in 2018, mainly in Carter Centre supported projects. It is not clear how many people this number of treatments relates as six monthly treatment was used in several sites. It also includes sites in Uganda where the vector has been eliminated and where ivermectin treatment was irrelevant for transmission elimination. They congratulate themselves that 2018 was the most successful year with stopping ivermectin treatment ever. This argument is misleading for the following reasons.

As onchocerciasis elimination is a new phase in Africa, it is not informative to make comparisons with past achievements. It would be more relevant to make comparisons with what is needed to be accomplished to meet the WHO objective of onchocerciasis elimination in at least 80% of endemic countries by the target date of 2025. Kim et al. [14] made detailed predictions of annual treatment needs and final years of treatment in all onchocerciasis endemic areas in Africa under different scenarios for onchocerciasis control, elimination and eradication. They predicted that the elimination scenario that was consistent with the above WHO elimination objective, would require treatment to have been stopped for 30 million people by 2017.

Tekle et al. [13] reported that the extensive epidemiological evaluations that were undertaken by APOC between 2009 and 2013 in 58 APOC project areas indicated that the results for 32 projects with 25 million people already met the criteria for stopping treatment or were close to elimination. Since these evaluations were carried out 6 years ago, most of the projects that were close to elimination would by now probably also meet the stopping treatment criteria. Hence, the evidence from APOC evaluations together with available evaluation data for OCP countries suggests that treatment can already be stopped for some 25–30 million people. Hence, although some progress has been made with stopping treatment, it represents only 10% of what probably is achievable and what is needed for adequate progress towards elimination by 2025.

There are multiple reasons for the insufficient progress. They include (i) the closure of APOC; (ii) problems with the failure to build on lessons learnt from OCP and APOC experience with onchocerciasis control in Africa; and (iii) the introduction of insufficiently reviewed and tested tools in an epidemiologically new and different environment. In our article we have elaborated on these problems and given several examples and references. In fact, as explained below, even the projects described by Richards et al. and Cupp et al. can be considered examples of such problems.

1. Duration and number of treatments

Both the Abu Hamed project in Sudan and the North Gondar project in Ethiopia mentioned by Richards et al. and Cupp et al. had very low precontrol endemicity levels for which the APOC methodology and evidence predicts 8 years of annual treatment would be sufficient for elimination. Instead, information on local endemicity levels was not taken into account and each of these projects apparently received around 20 rounds of treatment.

2. Criteria for elimination and stopping treatment

Richards et al. report that they have stopped treatment in Plateau and Nasarawa States in Nigeria after 24 rounds of annual treatment. Given the population size of this project, that is indeed a major achievement for which we congratulate them. It is interesting to note that currently, contrary to what Cupp et al. claim, globally, treatment has been stopped in more people who have received annual ivermectin treatment than in people who have been treated twice per year. Nevertheless we would like to note that a previous publication on the above project in which Dr. Richards was the senior author reported on the results of epidemiological and entomological evaluations done after 17 years of treatment in 5 LGA’s. In the publication it was noted that the elimination threshold for all epidemiological and entomological indicators had been reached except for OV 16 in children [15]. They recommended basing the decision to stop treatment on the prevalence of mf rather than on Ov16 since the prevalence of mf “was the clearest representation of active infection”, and concluded that ivermectin treatment could be stopped in the evaluated areas. That would have been consistent with APOC criteria and allowed cessation of treatment 7 years earlier than using the current OEPA protocol. At the end of the article they posed the question: “Is the OV16 antibody prevalence threshold of 0.1% (when used alone as an indicator of infection rates) too high of a standard for transmission interruption?”. We think that it is a good question to ask of an arbitrary threshold.

## Conclusions

The purpose of our article was not to object to changes in evaluation methods and decision-making criteria previously used in the African onchocerciasis control programmes. It was to express our concern that major lessons learned in over 40 years of successful onchocerciasis control in Africa were being ignored. Furthermore, it was to highlight that new methods and criteria which were developed in one epidemiological setting - for which there was no adequate scientific basis and which had not been adequately tested - were being introduced in clearly different epidemiological settings. Onchocerciasis control in Africa has always been highly scientific and innovative, involving continuous evaluation and evidence-based improvement. It is this open scientific approach we would like to see continued. We were therefore pleased to receive an informal response to our article from the WHO which admitted that the issues we raised in our article were largely valid and that an Onchocerciasis Technical Advisory Subgroup of the Technical Advisory Committee of the NTDs had been formed to address the issues and other shortcomings of the ongoing implementation of onchocerciasis elimination in Africa. We hope this initiative will result once more in an objective and inclusive scientific debate on the most appropriate strategies and tools for onchocerciasis elimination based on the best evidence and experience from all endemic regions in the world.

## Additional files

Additional file 1: Multilingual abstracts in the five official working languages of the United Nations. (PDF 207 kb)

## Data Availability

Not applicable.

## Abbreviations

APOC: African Programme for Onchocerciasis Control
MDA: Mass drug administration
OCP: Onchocerciasis Control Programme in West Africa
OEPA: Onchocerciasis Elimination Program for the Americas
WHO: World Health Organization
