# Supplementary material for: The need for evidence-based strategies and tools for onchocerciasis elimination in Africa
Source: Infect Dis Poverty. 2019 Jul 15;8:62. doi: 10.1186/s40249-019-0574-0 (PMC6628483; doi:10.1186/s40249-019-0574-0)
Supplement: Supplementary file 1 — Multilingual abstracts in the five official working languages of the United Nations. (PDF 207 kb) [file 40249_2019_574_MOESM1_ESM.pdf]

Translation of the abstract into the five official working languages of the United Nations

لقد أثبتت الحاجة إلى استراتيجيات وأدوات فاعليتها في التخلص من مرض العمى النهري (داء كلابية الذنب) في إفريقيا.

يانكوم دادزي، أوتشي ف. أمازيقو، بوكي أ. بوتن، أزودوغا سيكيتي

الملخص
في مقالة نشرت مؤخراً، تحدثنا عن إمكانية القضاء على مرض العمى النهري (داء كلابية الذنب) في إفريقيا بحلول عام 2025.
في هذه المقالة عبرنا عن قلقنا أن القضاء على هذا المرض قد لا ينجح بسبب عدم استخدام الدروس المستفادة من برامج السيطرة على مرض العمى النهري في إفريقيا، واستخدام استراتيجيات ووسائل أي بتت فاعليتها في أمريكا الجنوبية وأمريكا الشمالية. ريتشاردز وكاب مع باحثين آخرين رفضوا قلقنا وتحدثوا عن إنجازات حدثت مؤخراً بسبب توقف العلاج في بعض المناطق في إفريقيا.
في هذه المقالة، نتحدث عن حججهم التي قدموها بهذا الصدد و التي لم تكن مقنعه لنا. أشرنا إلى بعض العيوب العلمية في المفهوم الأمريكي للقضاء على المرض والتي تتطلب فترات طويلة من العلاج غير الضروري فضلاً عن إيقاف العلاج بطريقة عشوائية
لقد أظهرنا أن الإنجازات التي أشار إليها الباحثون لم تصل الى المبتغى، وهو القضاء على مرض العمى النهري بحلول عام 2025.
لقد ختمنا ردنا على الباحثين بالتاكيد على التزامنا بمناقشة أكثر موضوعيه وشمولية للبحث عن استراتيجيات ووسائل للقضاء على مرض العمى النهري.

Translated from English version into Arabic by Hamid Alahmari, Revised by Mohammad Jadallah, through

TRANSLATORS WITHOUT BORDERS

非洲消除盘尾丝虫病需要基于证据制定的策略和工具

Yankum Dadzie, Uche V. Amazigo, Boakye A. Boatin, Azodoga Sékétéli

摘要：
最近，我们发表了一篇文章中，讨论了 2025 年在非洲消除盘尾丝虫病的可行性。我们对消除工作可能因未能吸取非洲盘尾丝虫病控制项目的经验教训和引入美洲的战略和工具而受阻表示关切。Richards 和 Cupp 等团队写信驳斥了我们的担忧，并描述了近期在一些停止治疗的区域取得的成就。
作为回应，我们讨论了其中无法使我们信服的论点。我们指出美洲消除概念框架中存在若干科学问题，这些问题将导致过度治疗及主观的判断停止治疗标准。我们表明，最近取得的成就远远不足以实现在 2025 年消除非洲盘尾丝虫病。
最后，我们呼吁就消除盘尾丝虫病的策略和工具开展更加客观和具有包容性的讨论。

Translated from English version into Chinese by Peng Song, edited by Jin Chen

CDC CHINESE CENTER FOR DISEASE CONTROL AND PREVENTION
NATIONAL INSTITUTE OF PARASITIC DISEASES

Nécessité de stratégies et d'outils basés sur des données probantes pour éliminer l'onchocercose en Afrique

Yankum Dadzie, Uche V. Amazigo, Boakye A. Boatin, Azodoga Sékétéli

Résumé

Dans un récent article, nous discutions de la faisabilité de l'élimination de l'onchocercose en Afrique d'ici 2025. Nous craignions que cette élimination ne soit entravée par le manque d'exploitation des enseignements tirés des programmes africains de lutte contre l'onchocercose et par l'introduction de stratégies et outils employés dans les Amériques. Richards *et al.* et Cupp *et al.* ont écrit pour réfuter nos craintes et décrire les récents arrêts du traitement réussis dans certaines zones.

Leurs arguments ne nous ont pas convaincus, ainsi que nous l'exposons dans la présente réponse. Nous relevons plusieurs failles scientifiques dans le cadre conceptuel d'élimination employé aux Amériques, qui a mené à des périodes de traitement plus longues que nécessaire et à l'utilisation d'un seuil arbitraire pour l'arrêt du traitement. Nous démontrons que les succès récents sont loin d'être suffisants par rapport aux résultats qui seraient nécessaire pour éliminer l'onchocercose d'ici à 2025.

Nous concluons notre réponse en préconisant un débat plus objectif et inclusif sur les stratégies et outils pour l'élimination de l'onchocercose.

Translated from English version into French by Eva De Nadaï, Revised by Suzanne Assenat, through

TRANSLATORS WITHOUT BORDERS

Необходимость научно обоснованных стратегий и методов для ликвидации онхоцеркоза в Африке

Янкум Дадзи, Уче В. Амазиго, Боакье А. Боатин, Азодога Секетели

Аннотация

В недавней статье мы обсуждали возможность достижения ликвидации онхоцеркоза в Африке к 2025 году. Мы выразили обеспокоенность тем, что ликвидации может помешать невозможность применения опыта, приобретенного в ходе Африканской программы по борьбе с онхоцеркозом, и внедрение стратегий и методов из Америки. Ричардс и соавт. и Капп и соавт. обратились к тому, чтобы опровергнуть наше беспокойство, и описали недавние достижения по прекращению лечения в некоторых местностях.

В настоящем ответе мы обсуждаем их аргументы, которые нас не убеждают. Мы отмечаем несколько научных недостатков в американской концептуальной модели по ликвидации

заболевания, которая привела к более длительному, чем оно того требовало, лечению и к использованию произвольного порога для прекращения лечения.
Мы демонстрируем, что недавние достижения в значительной степени не соответствуют требованиям для ликвидации онхоцеркоза к 2025 году.
В заключении нашего ответа мы призываем к более объективной и всеобъемлющей дискуссии касательно стратегий и методов ликвидации онхоцеркоза.

Translated from English version into Russian by Alena Oleinik, Revised by Anna Kukharchuk, through

La necesidad de contar con estrategias y herramientas con base empírica para eliminar la oncocercosis en África

Yankum Dadzie, Uche V. Amazigo, Boakye A. Boatin, Azodoga Sékétéli

Resumen
En un artículo reciente discutimos la posibilidad de eliminar la oncocercosis en África para 2025. Expresamos preocupación por el hecho de que la eliminación pueda verse obstaculizada debido a la falta de aprovechamiento de las lecciones aprendidas en los programas de control de la oncocercosis en África y en la introducción de estrategias y herramientas provenientes de las Américas. Richards et al y Cupp et al han escrito para refutar nuestra preocupación y han descrito los recientes logros al interrumpir el tratamiento en ciertas zonas.
En esta respuesta, discutimos los argumentos que no consideramos convincentes. Señalamos varios defectos científicos en el marco conceptual americano respecto a la eliminación, que han resultado en períodos de tratamiento más largos de lo necesario y en el uso de un límite arbitrario para suspender el tratamiento. Demostramos que los logros recientes se sitúan considerablemente por debajo de lo necesario para lograr la eliminación de la oncocercosis para 2025.
Concluimos nuestra respuesta abogando por un debate sobre las estrategias y herramientas para la eliminación de la oncocercosis más objetivo e inclusivo.

Translated from English version into Spanish by Rosanna Lenci, Revised by Mayra León, through
